# Bioconvection Due to Gyrotactic Microorganisms in Couple Stress Hybrid Nanofluid Laminar Mixed Convection Incompressible Flow with Magnetic Nanoparticles and Chemical Reaction as Carrier for Targeted Drug Delivery through Porous Stretching Sheet

**DOI:** 10.3390/molecules26133954

**Published:** 2021-06-28

**Authors:** F. M. Alharbi, Muhammad Naeem, Muhammad Zubair, Muhammad Jawad, Wajid Ullah Jan, Rashid Jan

**Affiliations:** 1 Deanship of Combined First Year, Umm Al-Qura University Makkah, Mecca P.O. Box 715, Saudi Arabia; fmharbi@uqu.edu.sa (F.M.A.); mfaridoon@uqu.edu.sa (M.N.); 2 Department of Mathematics, Abdul Wali Khan University, Mardan 23200, Pakistan; zubair7colours@yahoo.com (M.Z.); mwajidmath@gmail.com (W.U.J.); 3 Department of Mathematics, University of Swabi, Swabi 94640, Pakistan; rashid_ash2000@yahoo.com

**Keywords:** nanoparticle mass, hemodynamics, chemical reaction, hybrid nanofluid, induced magnetic field, blood, gyrotactic microorganisms, drug delivery

## Abstract

In this paper, the steady electrically conducting hybrid nanofluid (CuO-Cu/blood) laminar-mixed convection incompressible flow at the stagnation-point with viscous and gyrotactic microorganisms is considered. Additionally, hybrid nanofluid flow over a horizontal porous stretching sheet along with an induced magnetic field and external magnetic field effects that can be used in biomedical fields, such as in drug delivery and the flow dynamics of the microcirculatory system. This investigation can also deliver a perfect view about the mass and heat transfer behavior of blood flow in a circulatory system and various hyperthermia treatments such as the treatment of cancer. The simple partial differential equations (PDEs) are converted into a series of dimensional ordinary differential equations (ODEs), which are determined using appropriate similarities variables (HAM). The influence of the suction or injection parameter, mixed convection, Prandtl number, buoyancy ratio parameter, permeability parameter, magnetic parameter, reciprocal magnetic prandtl number, bioconvection Rayleigh number, coupled stress parameter, thermophoretic parameter, Schmidt number, inertial parameter, heat source parameter, and Brownian motion parameter on the concentration, motile microorganisms, velocity, and temperature is outlined, and we study the physical importance of the present problem graphically.

## 1. Introduction

Bloodstream investigation in a human circulatory framework has developed amazing revenue in biotechnology and the world of medicine since most human diseases were caused by unsatisfactory supplies of blood to the lungs, veins, corridors, tissues, and systole stages. Numerous circulatory framework problems, including atherosclerosis embolism, aspiratory embolism and a blockage of blood supply in veins, nerves, and corridors, instigate a heart attack, stroke and ischemic thoracic inconvenience. By increased the blood temperature of 39 °C through 42 °C (hyperthermia) from average body temperature, the heart output doubles and the circulation increases. Nanomaterials have recently received a lot of attention in the field of biomedicine because of their useful applications, such as anticancer drug delivery, biosensing, antibacteria, and cell imaging, etc. Magnetic nanoparticles are extremely useful in magnetic drug targeting and magnetic resonance imaging agents, among other applications. Numerous studies have been conducted to determine the significance of nanoparticles in biological sciences. Chauhan and Tiwari [1] worked on non-Newtonian Herschel–Bulkley liquid and analyzed the heat transfer on blood movement in previous veins. They saw higher accuracy paper estimates generally decrease the blood velocity in veins. It is used for many positive therapies, such as cancers, cardiac drugs, and malignant development (Deussen [2] and Deniz [3]). With the aid of peristaltic and nanofluid, Bég and Tripathi [4] proposed the reconstruction of Mathematica’s bioengineering concept. Kothandapani and Prakash [5] found a heating source on an asymmetrical pointing channel on a non-Newtonian excessive digression nanofluid model. Akbar [6] examined delayed blood propagation of metal-based nanomaterial through the shaped stenotic route and explained nanomedicine applications. In Bhatti’s study [7], the properties and implementations of the vector viscosity blood clot model were investigated. The two-step model of peristalsis was considered in Dinarvand [8]. The constant laminar-blended mixture viscous and hybrid (CUO-Cu/blood) hybrid fluid flows near the plane stagnations on a level, permeable, linearly stretched board with an adjustable magnetic flux through a new nanoparticle and based liquid measurement [9]. Majee et al. conducted a methodical report on shaky blood progression with magnetic nanoparticles and is supposed to carry out the design of streams and nanoparticles in an infected blood vessel section that has atherosclerosis. Varshney [10] mathematically researched the pulsatile movement of blood going through a tightening vein, while the speed increase in the body is rambling. Jinga’s [11] research was conducted by combining a hybrid discrete component and a unit monitoring approach to calculate and stress the transmission behaviors of fractured crystalline rocks. The inspiration is the significance of insightful pressure impacts on the behavior, which are critical for the estimation of the environmental protection of many rock building projects, of the impurity transportation of broken crystalline rocks. Noorishad [12] presents a new means for the accurate testing of liquid stream lead in cracked permeable media, which is presented here. To do this, mechanical and liquid stream limits of both permeable and breakage media are used as part of an increase in Blot’s three-dimensional union hypothesis. Ellahi [13] introduced the peristaltic fluid stream between two coaxial cylinders of different forms and designs. The nanofluid consists of gold particles, while the pair of pressure fluids are filled as solvents. Choi and Eastman [14] created the term nanofluid, and this fluid is generated through a dilute inspection of solid particulate matter of 1–100 nm in constant fluids (oil, water, etc.). By the inclusion of ZnO, Cu, SiO_2_, TiO_2_, and Al_2_O_3_ nanopowders, the efficiency of the heat transfer of regular fluids has been greatly increased. In recent years, several investigators have discovered hypothetically and experimentally the characteristics of heat transfer from different nanoparticles in many manufacturing processes, development processes, and the application of renewable energy [15,16,17,18,19,20,21]. Researchers have developed various models for studying the Tiwari and Das model of nanofluids. Late in life, various researchers hypothetically and provisionally identified warmth motion attributes for several mechanical cycles, manufacturing, and environmentally friendly energy applications of different nanoparticles [15,16,17,18,19,20,21]. Specialists in nanofluids in which the Tiwari and Das model was presented with different models.

The macroscopic movement of fluid induces additional flexibility in swimming microorganisms, known as bioconvection, as a consequence of the 3-D variant in density over one region. The self-driving mobile microorganisms aim to boost the base fluid, creating a bioconvective stream in a specific direction. The travelling microorganisms are classified into different categories of chemical or oxytactical, gyrotactic, and negative gravitational characteristics. Nanoparticles are not self-regulated in comparison to mobile microorganisms, and the influence of the Brownian motion and the effect of thermophoresis is responsible for their motion. Nanofluid bioconvection is supposed to be feasible if the convergence of nanoparticles is low, and then, the choice for improved fluid thickness in the base is not sufficient. Basha [22] provided a mathematical response to the blood nanofluid fluid quality of a vehicle streaming across the plate, wedge, and stagnating stage. The effects of non-linear radiation, sticky dispersion, convinced magnetic field, and material reaction and the pertinence of the microorganism’s properties are evaluated. Bhatti [23] also studied the behavior of a changeable magnetic field and blood clot model using Jeffrey fluid nanoparticles and medication models. Ahmed [24] has regarded the magnetized, non-Darcy, permeable, laminar circulation of nanofluid and gyrotactic microorganisms. Kuznetsov [25] submitted nanoparticular suspension using the principle of Buongiorno for gyrotactic microorganisms. Raju [26] issued a mathematical study to determine Casson nanofluid stream liquid vehicles using gyrotactic microorganisms, which showed that adding the wedge point limits reduced the microorganism’s thickness. Raju and Rashad [27] addressed gyrotactic microorganisms’ impact on a chemical nanofluid flow over a vertical cylinder and noted that the amount of Rayleigh bioconvection increases the density of microorganisms. Liao (1992) [28] has observed that this technique is fast convergent to the approximate solution, and it is the best fit for the solution of non-linear problems. We considered an electrically guided coupled pressure crossover (CuO-Cu/blood) nanofluid stream comprised of gyrotactic microorganisms pushed near the plane stagnation-point over a level, permeable, extending layer alongside an outer attractive field and prompted attractive field impacts in the current study. This investigation will also provide a good image of the temperature and mass exchange activity of blood in a circulatory system, as well as various hyperthermia treatments, such as cancer care. The Buongiorno model was used to demonstrate thermophoresis and Brownian dispersion. The sheet is permeable, and the surface of the stretching sheet has an injection effect. Our model is mathematically formulated by deriving the governing equations and applying sufficient similarity transformations. Using the Homotopy Analysis process, we can solve our modeled problem.

### 1.1. Problem Mathematical Modeling

**Figure 1:**
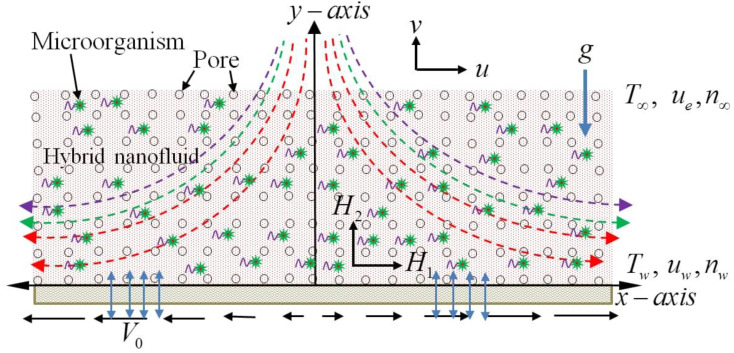
Two-dimensional problem and coordinate system of the Schematic diagram.

We are assuming the continuously laminar-varied convection compact viscous and the electrically guided coupling pressure Darcy–Forchheimer CuO-Cu/Blood hybrid nanofluid fluids and heat close to the stagnation point on a smooth, directly extending the plate below an exterior magnetic flux, as defined in Figure 1. The thermophoresis and Brownian diffusion effects were analyzed using the Buongiorno model. The sheet is permeable, and the stretching sheet’s surface has an injection effect. Moreover, the suction/injection velocity of the sheet is $V_{0}$, while the linear stretching velocity is $u_{w}=cx$. Furthermore, the sheet’s temperature depends on the stagnation point, i.e., $T_{w}=T_{\infty }+T_{0}\frac{x}{l}\;$. The control of the boundary layer and external magnetic flux towards *x*- direction can be represented by $u_{e}=ax$ and $H_{e}\left(x\right)=H_{0}\frac{x}{l}$. We stress that this evaluation is a characteristic length l of the uniform magnetic field $H_{0}$ in the upstream infinity. Table 1 display the thermophysical properties of *c_p_*, *ρ*, *k* and *β*. The following principles can be expressed in the following terms: simple non-linear PDEs.

(1) $$\frac{\partial u}{\partial x}\;\;\;+\;\;\;\frac{\partial v}{\partial y}=0,$$

(2) $$\frac{\partial H_{1}}{\partial x}\;\;\;+\;\;\;\frac{\partial H_{2}}{\partial y}=0,$$

(3) $$\begin{array}{l} u\frac{\partial u}{\partial x}\;\;\;+\;\;\;v\frac{\partial u}{\partial y}-\frac{\mu_{e}}{4\pi \rho_{hnf}}\left(H_{2}\frac{\partial H_{1}}{\partial x}\;\;\;+\;\;H_{2}\;\frac{\partial H_{1}}{\partial y}\right)=\mu_{e}\frac{d\mu_{e}}{dx}\;-\frac{\mu_{e}}{4\pi \rho_{hnf}}H_{e}\frac{dH_{e}}{dx}+\frac{\mu_{hnf}}{\rho_{hnf}}\frac{\partial^{2}u}{\partial y^{2}}\\ -\frac{\mu_{hnf}}{\rho_{hnf}}\frac{u}{k^{*}}-\frac{\eta_{0}}{\rho_{hnf}}\frac{\partial^{4}u}{\partial y^{4}}+-\frac{\left(\rho_{m}-\rho_{f}\right)\left(n-n_{\infty }\right)}{\rho_{hnf}}g-\frac{\left(\rho_{p}-\rho_{f}\right)\left(C-C_{\infty }\right)}{\rho_{hnf}}g+\frac{\left(1-C_{\infty }\right)\beta \rho_{f}\left(T-T_{\infty }\right)g}{\rho_{hnf}}, \end{array}$$

(4) $$u\frac{\partial H_{1}}{\partial x}\;\;\;+\;\;v\frac{\partial H_{1}}{\partial y}-H_{1}\frac{\partial u}{\partial x}-H_{2}\frac{\partial u}{\partial y}=\eta_{0}\frac{\partial^{2}H_{1}}{\partial y^{2}},$$

(5) $$u\frac{\partial T}{\partial x}\;\;\;+\;\;\;v\frac{\partial T}{\partial y}=\frac{k_{hnf}}{{\left(\rho c_{p}\right)}_{hnf}}\frac{\partial^{2}T}{\partial y^{2}}+\tau \left[D_{B}\left(\frac{\partial C}{\partial y}\frac{\partial T}{\partial y}\right)+\frac{D_{T}}{{Τ}_{\infty }}{\left(\frac{\partial T}{\partial y}\right)}^{2}\right]+\frac{Q}{{\left(\rho c_{p}\right)}_{hnf}}\left(T-T_{\infty }\right),$$

(6) $$u\frac{\partial C}{\partial x}\;\;\;+\;\;\;v\frac{\partial C}{\partial y}=D_{B}\frac{\partial^{2}C}{\partial y^{2}}+\left(\frac{D_{T}}{{Τ}_{\infty }}\right)\frac{\partial^{2}T}{\partial y^{2}}-K_{r}\left(C-C_{\infty }\right),$$

(7) $$uN_{x}+vN_{y}+\frac{b^{*}W_{c}}{\left(C_{w}-C_{\infty }\right)}{\left(NC_{y}\right)}_{y}=D_{N}N_{yy},$$

Following to the boundary conditions:

(8) $$\begin{array}{l} u=u_{w}=cx,v=-V_{0},\frac{\partial H_{1}}{\partial y}=H_{2}=0,T=T_{w}=T_{\infty }+T_{0}\frac{x}{l}\;,\;D_{B}\frac{\partial C}{\partial y}+\frac{D_{T}}{{Τ}_{\infty }}\frac{\partial T}{\partial y},N=N_{w}\;\;at\;y=0,\\ u=\mu_{e}=ax,H_{1}=H_{e}=H_{0}\frac{x}{l},T=T_{\infty },C=C_{\infty },\;N=N_{\infty }\;\;\;\;\;\;\;\;\;\;\;\;\;\;\;\;\;\;\;\;\;\;\;\;\;\;\;\;\;\;\;\;\;\;\;\;\;\;\;\;\;\;\;\;\;at\;\;y=\infty . \end{array}$$

where the position temperature $T_{0}$, the atmospheric temperature ${Τ}_{\infty }$, magnetic permeability $\mu_{e}$, u and v, alongside x− and y− axes $H_{1}$ and $H_{2}$ are the velocity units with persuaded components of the magnetic field on each. The dimension $k^{*}$ is the magnetic absorption potential of the permeable intermediate. Diffusivity g is gravity acceleration, temperature is T, β is the thermal growth volumetric constant, and Q is the heat generation volumetric rate/absorption. $\rho_{hnf}$, $\mu_{hnf}$, ${\left(\rho c_{p}\right)}_{hnf}$ and $k_{hnf}$ are the density, viscosity, heat power volumetric, and hybrid nanofluid thermal conductivity calculated, respectively. These can be seen from Table 2.

**Figure 2:**
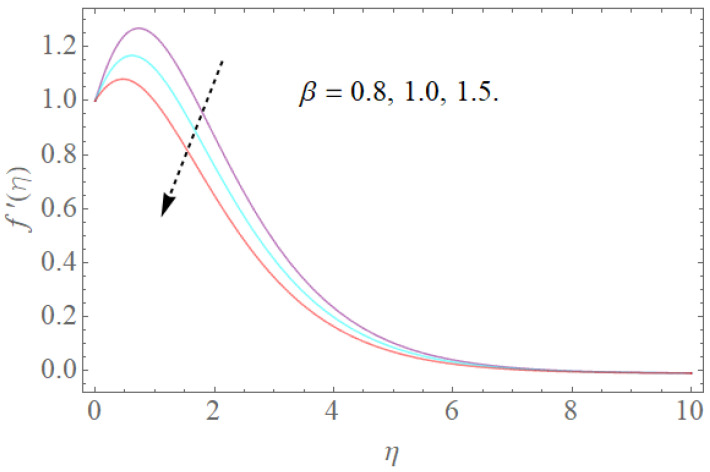
The effect of β on $f^{\prime }\left(\eta \right)$ when $k_{1}=0.3,K=0.7,R_{b}=0.4,N_{r}=2.0,\lambda =1.1$.

As we note, the experimental type factor of the nanoparticles seen in Figure 2 is the classic Hamilton–Crosser approximate for real thermal conductivity.

It is worthwhile to mention here that, we suggest ϕ, $\rho_{s}$ and ${\left(c_{p}\right)}_{s}$ as the equivalent nanoparticle volume fraction, the equivalent density of nanoparticles and the equivalents pecific heat at constant pressure of nanoparticles, respectively. Moreover, respectively, $\phi_{1}$ and $\phi_{2}$ are the 1st and 2nd nanoparticles’ volume fraction of these compatible formulae:

(9) $$\rho_{s}=\frac{\left(\rho_{1}\times w_{1}\right)+\left(\rho_{2}\times w_{2}\right)}{w_{1}+w_{2}}$$

(10) $${\left(\rho c_{p}\right)}_{s}=\frac{\left({\left(c_{p}\right)}_{1}\times w_{1}\right)+\left({\left(c_{p}\right)}_{2}\times w_{2}\right)}{w_{1}+w_{2}}$$

**Table 2:** Adapted frameworks and thermophysical properties for the hybrid nanofluid.

Property	Hybrid Nanofluid
Viscosity (*μ*)	$\frac{\mu_{f}}{{\left(1-\phi \right)}^{2.5}}$
Volumetric heat capacity (*ρ*)	$\left(1-\phi \right)\rho_{f}+\phi \rho_{s}$
Volumetric heat capacity (*ρc_p_*)	$\left(1-\phi \right){\left(\rho c_{p}\right)}_{f}+\phi {\left(\rho c_{p}\right)}_{s}$
Thermal conductivity (*k*)	$\frac{k_{2}+\left(n_{2}-1\right)k_{f}-\left(n_{2}-1\right)\phi_{1}\left(k_{f}-k_{2}\right)}{k_{2}+\left(n_{2}-1\right)k_{nf}+\phi_{2}\left(k_{nf}-k_{2}\right)}$ $\times \frac{k_{1}+\left(n_{1}-1\right)k_{f}-\left(n_{1}-1\right)\phi_{1}\left(k_{f}-k_{1}\right)}{k_{1}+\left(n_{1}-1\right)k_{nf}+\phi_{2}\left(k_{nf}-k_{1}\right)}\times k_{f}$; $k_{nf}=\frac{k_{1}+\left(n_{1}-1\right)k_{f}-\left(n_{1}-1\right)\phi_{1}\left(k_{f}-k_{1}\right)}{k_{1}+\left(n_{1}-1\right)k_{nf}+\phi_{2}\left(k_{nf}-k_{1}\right)}\times k_{f}$
	$\phi_{1}=\frac{\frac{w_{1}}{\rho_{1}}}{\frac{w_{1}}{\rho_{1}}+\frac{w_{2}}{\rho_{2}}+\frac{w_{f}}{\rho_{f}}},$ (11)
	$\phi_{2}=\frac{\frac{w_{2}}{\rho_{2}}}{\frac{w_{1}}{\rho_{1}}+\frac{w_{2}}{\rho_{2}}+\frac{w_{f}}{\rho_{f}}},$ (12)
	$\phi =\phi_{1}+\phi_{2},$ (13)

More, in Equations (12)–(15), a group of similar variables are introduced, and $w_{1}$, $w_{2}$ and $w_{f}$ are the 1st and 2nd nanoparticles and the base fluid masses, respectively.

(14) $$\begin{array}{l} \eta ={\left(\frac{c}{\nu_{f}}\right)}^{1/2}y,\psi ={\left(c\nu_{f}\right)}^{1/2}xf\left(\eta \right),H_{1}=\frac{H_{0}x}{l}g^{\prime }\left(\eta \right),\\ H_{2}=-{\left(\frac{\nu_{f}}{cl^{2}}\right)}^{1/2}H_{0}g\left(\eta \right),\theta (\eta )=\frac{Τ-{Τ}_{\infty }}{{Τ}_{w}-{Τ}_{\infty }},\phi (\eta )=\frac{C-C_{\infty }}{C_{w}-C_{\infty }}. \end{array}$$

Putting Equation (17) into dimensional administering, Equations (1)–(4), (6), and (11) tips to accomplish these dimensionless non-linear overseeing ODEs.

(15) $$KA_{1}f^{v}+f^{\prime \prime \prime }+\frac{1}{k_{1}}f^{\prime }-\frac{\lambda \rho_{f}}{\rho_{hnf}}\left[\theta -N_{r}\phi -R_{b}\xi \right]+A_{1}\left({\left(\frac{a}{c}\right)}^{2}+ff^{\prime \prime }-{f^{\prime }}^{2}\right)+\beta A_{2}\left({g^{\prime }}^{2}-gg^{\prime \prime }-1\right)=0,$$

(16) $$\Lambda g^{\prime \prime \prime }+fg^{\prime \prime }-f^{\prime \prime }g=0,$$

(17) $$\theta^{\prime \prime }+\frac{k_{f}}{k_{hnf}}A_{4}\mathrm{Pr}\theta^{\prime }+\frac{k_{f}}{k_{hnf}}\alpha \theta +\frac{{\left(kc_{p}\right)}_{f}}{k_{hnf}}A_{4}\mathrm{Pr}\left[N_{b}\theta^{\prime }\phi^{\prime }+N_{t}{\theta^{\prime }}^{2}\right]=0,$$

(18) $$\Phi^{\prime \prime }+Scf\Phi^{\prime }+\frac{N_{t}}{N_{b}}\theta^{\prime \prime }-A\Phi =0,$$

(19) $$\xi^{\prime \prime }-P_{e}\left(\left(\xi +\sigma_{1}\right)\phi^{\prime \prime }+\xi^{\prime }\phi^{\prime }\right)-\mathrm{Pr}L_{b}f\xi^{\prime }=0.$$

$$\begin{array}{l} A_{1}=A_{2}\times \left(1-\frac{\frac{w_{1}}{\rho_{1}}+\frac{w_{2}}{\rho_{2}}}{\frac{w_{1}}{\rho_{1}}+\frac{w_{2}}{\rho_{2}}+\frac{w_{f}}{\rho_{f}}}+\frac{\frac{w_{1}}{\rho_{1}}+\frac{w_{2}}{\rho_{2}}}{\frac{w_{1}}{\rho_{1}}+\frac{w_{2}}{\rho_{2}}+\frac{w_{f}}{\rho_{f}}}\frac{\rho_{s}}{\rho_{f}}\right),A_{2}=\left(1-\frac{\frac{w_{1}}{\rho_{1}}+\frac{w_{2}}{\rho_{2}}}{\frac{w_{1}}{\rho_{1}}+\frac{w_{2}}{\rho_{2}}+\frac{w_{f}}{\rho_{f}}}\right)\\ A_{3}=A_{2}\left[\frac{\frac{w_{2}}{\rho_{2}}}{\frac{w_{1}}{\rho_{1}}+\frac{w_{2}}{\rho_{2}}+\frac{w_{f}}{\rho_{f}}}\left\{\left(1-\frac{\frac{w_{1}}{\rho_{1}}}{\frac{w_{1}}{\rho_{1}}+\frac{w_{2}}{\rho_{2}}+\frac{w_{f}}{\rho_{f}}}\right)+\frac{\frac{w_{1}}{\rho_{1}}}{\frac{w_{1}}{\rho_{1}}+\frac{w_{2}}{\rho_{2}}+\frac{w_{f}}{\rho_{f}}}\frac{{\left(\rho \beta \right)}_{1}}{{\left(\rho \beta \right)}_{f}}+\frac{\frac{w_{2}}{\rho_{2}}}{\frac{w_{1}}{\rho_{1}}+\frac{w_{2}}{\rho_{2}}+\frac{w_{f}}{\rho_{f}}}\frac{{\left(\rho \beta \right)}_{2}}{{\left(\rho \beta \right)}_{f}}\right\}\right]\\ A_{4}=\left(1-\frac{\frac{w_{1}}{\rho_{1}}+\frac{w_{2}}{\rho_{2}}}{\frac{w_{1}}{\rho_{1}}+\frac{w_{2}}{\rho_{2}}+\frac{w_{f}}{\rho_{f}}}+\frac{\frac{w_{1}}{\rho_{1}}+\frac{w_{2}}{\rho_{2}}}{\frac{w_{1}}{\rho_{1}}+\frac{w_{2}}{\rho_{2}}+\frac{w_{f}}{\rho_{f}}}\frac{{\left(\rho c_{p}\right)}_{s}}{{\left(\rho c_{p}\right)}_{f}}\right) \end{array}$$

Comparably, replacing Equation (17) with Equation (7) provides one with the following dimensionless boundary conditions:

(20) $$\begin{array}{l} f\left(0\right)=s,f^{\prime }\left(0\right)=1,g\left(0\right)=g^{\prime \prime }\left(0\right)=0,\theta \left(0\right)=1,\phi \left(0\right)=1,\xi \left(0\right)=1,\\ f^{\prime }\left(\infty \right)\to \frac{a}{c},g^{\prime }\left(\infty \right)\to 1,\theta \left(\infty \right)\to 0,\phi \left(\infty \right)\to 0,\xi \left(\infty \right)\to 0. \end{array}$$

Obviously, primes signify the separation regarding η. In the current issue, administering parameters, for example, Prandtl number (Pr), suction or injection parameter (s), penetrability boundary $\left(k_{1}\right)$, magnetic boundary (β), blended convection or lightness boundary (λ), corresponding magnetic Prandtl number (Λ), inertial boundary $\left(F_{1}\right)$, thermophoretic boundary $\left(N_{t}\right)$, Brownian movement boundary $\left(N_{b}\right)$, coupled pressure boundary (K), Schmidt number (Sc), and heat source boundary (α), are characterized as:

(21) Pr=νfαf,s=V0(cνf)1/2,k1=ck*νf,β=μe4πρf(H0lc)2,α=Qνfckf,Λ=η0νf,F1=υfcbkfλ=GrxRex2,Grx=gβf(T−T∞)x3(νf)2,Rex=uwxνf=x2cνf,R=4σ1T∞3k*kf,Nb=τDB(Cw−C∞)νf,Nt=τDT(Τs−Τ0)νfΤ0,Sc=νfDBNr=(ρp−ρf)ΔCρfβ(1−C∞)ΔT,Pe=b*WcDm,Lb=αDN,σ1=N∞Nw−N∞,Rb=(ρm−ρf)gγΔnρfβ(1−C∞)ΔT.

where the local Grashof number, bioconvection Rayleigh number, bioconvection Peclet number, bioconvection Lewis number, buoyancy ratio parameter, concentration difference parameter, and the local Reynolds number, respectively, are denoted by the following symbols $Gr_{x}\;,R_{b},P_{e},L_{b},N_{r},\sigma_{1}$, and  Rex. The suction and injection should be noted and correlate to the suction.

### 1.2. Physical Quantities of Interest

For the above model, the local Nusselt number $\left(Nu_{x}\right)$, local Sherwood number $\left(Sh_{x}\right)$, and skin friction coefficient $\left(c_{fx}\right)$ are clear as follows:

(22) $$c_{fx}=\frac{\tau_{w}}{\rho_{f}u_{w}^{2}},Nu_{x}=\frac{xq_{w}}{k_{f}\left(T-T_{\infty }\right)},Sh_{x}=\frac{xq_{m}}{D_{B}\left(C-C_{\infty }\right)}$$

where

(23) $$\tau_{w}=\mu_{hnf}{\left(u_{y}\right)}_{y=0},q_{w}={-k_{hnf}\left(\left(T_{y}\right)-q_{r}\right)|}_{y=0},q_{m}={-D_{B}\left(C_{y}\right)|}_{y=0}\;$$

From above, we have the dimensional form as

(24) [Rex]12Cf=(1−w1ρ1+w2ρ2w1ρ1+w2ρ2+wfρf)f″(0),[Rex]−12Nux=−khnfkfθ′(0),[Rex]−12Sh=−ϕ′(0),NnxRex−1/2=−ξ′(0).

### 1.3. Solution by HAM

Boundary conditions (Equation (20)) with Equations (15)–(19) have been resolved via HAM. Mathematica programming is utilized for this objective.

(25) $$L_{\overset{⌢}{f}}(\overset{⌢}{f})={\overset{⌢}{f}}^{v},L_{\overset{⌢}{g}}(\overset{⌢}{g})={\overset{⌢}{g}}^{\prime \prime \prime },L_{\overset{⌢}{\theta }}(\overset{⌢}{\theta })={\overset{⌢}{\theta }}^{\prime \prime }{,L}_{\overset{⌢}{\Phi }}(\overset{⌢}{\Phi })={\overset{⌢}{\Phi }}^{\prime \prime }\;,L_{\overset{⌢}{\xi }}(\overset{⌢}{\xi })={\overset{⌢}{\xi }}^{\prime \prime },$$

The linear operators are presented as:

(26) $$\begin{array}{l} L_{\overset{⌢}{f}}(e_{1}+e_{2}\eta +e_{3}\eta^{2}+e_{4}\eta^{3}+e_{5}\eta^{4})=0,L_{\overset{⌢}{g}}(e_{6}+e_{7}\eta +e_{8}\eta^{2})=0,\\ L_{\overset{⌢}{\theta }}(e_{9}+e_{10}\eta )=0,L_{\overset{⌢}{\Phi }}(e_{11}+e_{12}\eta )=0\;,L_{\overset{⌢}{\xi }}(e_{13}+e_{14}\eta )=0 \end{array}$$

The non-linear operatives are chosen as $N_{\overset{⌢}{f}},N_{\overset{⌢}{g}},N_{\overset{⌢}{\theta }}\;and\;\;N_{\overset{⌢}{\phi }}\;$ and are identified in the following systems:

(27) $$\begin{array}{l} \;N_{\overset{⌢}{f}}\;\left[\overset{⌢}{f}(\eta ;\;\;\zeta ),\overset{⌢}{g}(\eta ;\;\;\zeta ),\overset{⌢}{\theta }(\eta ;\;\;\zeta ),\overset{⌢}{\Phi }(\eta ;\;\;\zeta ),\overset{⌢}{\xi }(\eta ;\;\;\zeta )\right]=\;\;KA_{1}{\overset{⌢}{f}}_{\eta \eta \eta \eta \eta }+{\overset{⌢}{f}}_{\eta \eta \eta }+\frac{1}{k_{1}}{\overset{⌢}{f}}_{\eta }\\ -\frac{\lambda \rho_{f}}{\rho_{hnf}}\left[\overset{⌢}{\theta }-N_{r}\overset{⌢}{\Phi }-R_{b}\overset{⌢}{\xi }\right]\;+A_{1}\left({\left(\frac{a}{c}\right)}^{2}+\overset{⌢}{f}{\overset{⌢}{f}}_{\eta \eta }-{\overset{⌢}{f}}_{\eta }{}^{2}\right)+\beta A_{2}\left({\overset{⌢}{g}}_{\eta }^{2}-\overset{⌢}{g}{\overset{⌢}{g}}_{\eta \eta }-1\right), \end{array}$$

(28) $$N_{\overset{⌢}{g}}\;\left[\overset{⌢}{f}(\eta ;\;\;\zeta ),\overset{⌢}{g}(\eta ;\;\;\zeta )\right]=\;\Lambda {\overset{⌢}{g}}_{\eta \eta \eta }+\overset{⌢}{f}{\overset{⌢}{g}}_{\eta \eta }-{\overset{⌢}{f}}_{\eta \eta }\overset{⌢}{g},$$

(29) $$\begin{array}{l} N_{\overset{⌢}{\theta }}\left[\overset{⌢}{\theta }(\eta ;\zeta ),\overset{⌢}{\Phi }(\eta ;\zeta )\right]=\left(1+\frac{4}{3}R\right){\overset{⌢}{\theta }}_{\eta \eta }+\frac{k_{f}}{k_{hnf}}A_{4}\mathrm{Pr}{\overset{⌢}{\theta }}_{\eta }+\frac{k_{f}}{k_{hnf}}\alpha \overset{⌢}{\theta }\\ +\frac{{\left(kc_{p}\right)}_{f}}{k_{hnf}}A_{4}\mathrm{Pr}\left(N_{b}{\overset{⌢}{\theta }}_{\eta }{\overset{⌢}{\phi }}_{\eta }+N_{t}{\overset{⌢}{\theta }}_{\eta }{}^{2}\right), \end{array}$$

(30) $$N_{\overset{⌢}{\Phi }}\left[\overset{⌢}{\Phi }(\eta ;\zeta ),\overset{⌢}{f}(\eta ;\zeta ),\overset{⌢}{\theta }(\eta ;\zeta )\right]={\overset{⌢}{\Phi }}_{\eta \eta }-Sc\overset{⌢}{f}{\overset{⌢}{\Phi }}_{\eta }+\frac{N_{t}}{N_{b}}{\overset{⌢}{\theta }}_{\eta \eta }-A\overset{⌢}{\Phi },$$

(31) $$N_{\overset{⌢}{\xi }}\left[\overset{⌢}{\xi }(\eta ;\zeta ),\overset{⌢}{f}(\eta ;\zeta ),\overset{⌢}{\xi }(\eta ;\zeta )\right]={\overset{⌢}{\xi }}_{\eta \eta }-P_{e}\left(\left(\overset{⌢}{\xi }+\sigma_{1}\right){\overset{⌢}{\Phi }}_{\eta \eta }+{\overset{⌢}{\xi }}_{\eta }{\overset{⌢}{\Phi }}_{\eta }\right)-\mathrm{Pr}L_{b}\overset{⌢}{f}{\overset{⌢}{\xi }}_{\eta }.$$

Moreover, BCs are:

(32) $$\begin{array}{l} {\frac{\partial \overset{⌢}{f}(\eta ;\zeta )}{\partial \eta }|}_{\eta =0}=1,\;{\overset{⌢}{f}(\eta ;\zeta )|}_{\eta =0}=s,{{\frac{\partial^{2}\overset{⌢}{g}(\eta ;\zeta )}{\partial^{2}\eta }|}_{\eta =0}=\overset{⌢}{g}(\eta ;\zeta )|}_{\eta =0}=0,{\overset{⌢}{\theta }(\eta ;\zeta )|}_{\eta =0}=1,{\overset{⌢}{\Phi }(\eta ;\zeta )|}_{\eta =0}=1,{\overset{⌢}{\xi }(\eta ;\zeta )|}_{\eta =0}\\ {\frac{\partial \overset{⌢}{f}(\eta ;\zeta )}{\partial \eta }|}_{\eta =\infty }=\frac{a}{c},{\frac{\partial \overset{⌢}{g}(\eta ;\zeta )}{\partial \eta }|}_{\eta =\infty }=1,{\overset{⌢}{\theta }(\eta ;\zeta )|}_{\eta =\infty }=0,{\overset{⌢}{\Phi }(\eta ;\zeta )|}_{\eta =\infty }=0,{\overset{⌢}{\xi }(\eta ;\zeta )|}_{\eta =\infty }=0. \end{array}$$

Here, ζ is the embedding parameter. ζ∈[0,1] is used to standardize the convergence of the solution of $\hbar_{\overset{⌢}{f}},\hbar_{\overset{⌢}{g}}$, $\hbar_{\overset{⌢}{\theta }}$ and $\hbar_{\overset{⌢}{\phi }}$ By choosing ζ=0 and ζ=1 [27], we have:

(33) $$\overset{⌢}{f}\left(\eta ;1\right)=\overset{⌢}{f}(\eta ),\overset{⌢}{g}\left(\eta ;1\right)=\overset{⌢}{g}(\eta )\;\;\;\;\overset{⌢}{\theta }(\eta ;1)=\overset{⌢}{\theta }(\eta )\;,\;\;\;\;\;\overset{⌢}{\Phi }(\eta ;1)=\overset{⌢}{\Phi }(\eta ),\overset{⌢}{\xi }(\eta ;1)=\overset{⌢}{\xi }(\eta ),$$

Develop Taylor’s series for $\overset{⌢}{f}(\eta ;\zeta ),\overset{⌢}{g}(\eta ;\zeta ),\overset{⌢}{\theta }(\eta ;\zeta ),\overset{⌢}{\Phi }(\eta ;\zeta )$, and $\overset{⌢}{\xi }(\eta ;\zeta )$ about the point ζ=0:

(34) $$\begin{array}{l} \overset{⌢}{f}(\eta ;\zeta )={\overset{⌢}{f}}_{0}(\eta )+\sum_{n=1}^{\infty }{\overset{⌢}{f}}_{n}(\eta )\zeta^{n}\\ \overset{⌢}{g}(\eta ;\zeta )={\overset{⌢}{g}}_{0}(\eta )+\sum_{n=1}^{\infty }{\overset{⌢}{g}}_{n}(\eta )\zeta^{n}\\ \overset{⌢}{\theta }(\eta ;\zeta )={\overset{⌢}{\theta }}_{0}(\eta )+\sum_{n=1}^{\infty }{\overset{⌢}{\theta }}_{n}(\eta )\zeta^{n}\\ \overset{⌢}{\Phi }(\eta ;\zeta )={\overset{⌢}{\Phi }}_{0}(\eta )+\sum_{n=1}^{\infty }{\overset{⌢}{\Phi }}_{n}(\eta )\zeta^{n}\\ \overset{⌢}{\xi }(\eta ;\zeta )={\overset{⌢}{\xi }}_{0}(\eta )+\sum_{n=1}^{\infty }{\overset{⌢}{\xi }}_{n}(\eta )\zeta^{n} \end{array}$$

(35) $$\begin{array}{l} {\overset{⌢}{f}}_{n}(\eta )={\frac{1}{n!}\frac{\partial \overset{⌢}{f}\left(\eta ;\zeta \right)}{\partial \eta }|}_{p=0},{\overset{⌢}{g}}_{n}(\eta )={\frac{1}{n!}\frac{\partial \overset{⌢}{g}\left(\eta ;\zeta \right)}{\partial \eta }|}_{p=0},\;\;\;\;\;\;\;\;\;{\overset{⌢}{\theta }}_{n}(\eta )={\frac{1}{n!}\frac{\partial \overset{⌢}{\theta }\left(\eta ;\zeta \right)}{\partial \eta }|}_{p=0},\;\;\\ \;\;\;\;{\overset{⌢}{\Phi }}_{n}(\eta )={\frac{1}{n!}\frac{\partial \overset{⌢}{\Phi }(\eta ;\zeta )}{\partial \eta }|}_{p=0},{\overset{⌢}{\xi }}_{n}(\eta )={\frac{1}{n!}\frac{\partial \overset{⌢}{\xi }(\eta ;\zeta )}{\partial \eta }|}_{p=0}. \end{array}$$

Moreover, BCs are:

(36) $$\begin{array}{l} \overset{⌢}{f}\left(0\right)=s,\overset{⌢}{f^{\prime }}\left(0\right)=1,\overset{⌢}{g}\left(0\right)=\overset{⌢}{g^{\prime \prime }}\left(0\right)=0,\overset{⌢}{\theta }\left(0\right)=1,\overset{⌢}{\Phi }\left(0\right)=1,\\ \overset{⌢}{f^{\prime }}\left(\infty \right)\to \frac{a}{c},\overset{⌢}{g}\left(\infty \right)\to 1,\overset{⌢}{\theta }\left(\infty \right)\to 0,\overset{⌢}{\Phi }\left(\infty \right)\to 0. \end{array}$$

Now:

(37) $$\begin{array}{l} {ℜ}_{n}^{\overset{⌢}{f}}\left(\eta \right)=\frac{\mu_{hnf}/\mu_{f}}{\rho_{hnf}/\rho_{f}}{\overset{⌢}{f}}_{n-1}^{v}+{\overset{⌢}{f}}_{n-1}^{\prime \prime \prime }+\frac{1}{k_{1}}{\overset{⌢}{f}}_{n-1}^{\prime }-\frac{\lambda \rho_{f}}{\rho_{hnf}}\left[{\overset{⌢}{\theta }}_{n-1}-N_{r}{\overset{⌢}{\Phi }}_{n-1}-R_{b}{\overset{⌢}{\xi }}_{n-1}\right]\\ +A_{1}\left({\left(\frac{a}{c}\right)}^{2}+\sum_{j=0}^{w-1}{\overset{⌢}{f}}_{w-1-j}{\overset{⌢}{f}}_{j}^{\prime \prime }-{{\overset{⌢}{f}}^{\prime }}_{n-1}^{2}\right)+\beta A_{2}\left({{\overset{⌢}{g}}^{\prime }}_{n-1}^{2}-\sum_{j=0}^{w-1}{\overset{⌢}{g}}_{w-1-j}{\overset{⌢}{g}}_{j}^{\prime \prime }-1\right), \end{array}$$

(38) $${ℜ}_{n}^{\overset{⌢}{g}}(\eta )=\Lambda {\overset{⌢}{g}}_{n-1}^{\prime \prime \prime }+\sum_{j=0}^{w-1}{\overset{⌢}{f}}_{w-1-j}{\overset{⌢}{g}}_{j}^{\prime \prime }-2\sum_{j=0}^{w-1}{\overset{⌢}{f}}_{w-1-j}^{\prime \prime }{\overset{⌢}{g}}_{j},$$

(39) $$\begin{array}{l} {ℜ}_{n}^{\overset{⌢}{\theta }}(\eta )=\left(1+\frac{4}{3}R\right)\left({\overset{⌢}{\theta }}_{n-1}^{\prime \prime }\right)+\frac{k_{f}}{k_{hnf}}A_{4}\mathrm{Pr}{\overset{⌢}{\theta }}_{n-1}^{\prime }+\frac{k_{f}}{k_{hnf}}\alpha {\overset{⌢}{\theta }}_{n-1}+\\ \frac{{\left(kc_{p}\right)}_{f}}{k_{hnf}}A_{4}\mathrm{Pr}\left(N_{b}\sum_{j=0}^{w-1}{\overset{⌢}{\theta }}_{w-1-j}^{\prime }{\overset{⌢}{\phi }}_{j}^{\prime }+N_{t}{{\overset{⌢}{\theta }}^{\prime \prime }}_{n-1}^{2}\right), \end{array}$$

(40) $${ℜ}_{n}^{\overset{⌢}{\Phi }}\left(\eta \right)={\overset{⌢}{\Phi }}_{n-1}^{\prime \prime }-Sc\sum_{j=0}^{w-1}{\overset{⌢}{f}}_{w-1-j}{\overset{⌢}{\Phi }}_{j}^{\prime }+\frac{N_{t}}{N_{b}}{\overset{⌢}{\theta }}_{n-1}^{\prime \prime }-A{\overset{⌢}{\Phi }}_{n-1},$$

(41) $${ℜ}_{n}^{\overset{⌢}{\xi }}\left(\eta \right)={\overset{⌢}{\xi }}_{n-1}^{\prime \prime }-P_{e}\left(\sum_{j=0}^{w-1}\left({\overset{⌢}{\xi }}_{w-1-j}+\sigma_{1}\right){{\overset{⌢}{\phi }}^{\prime \prime }}_{j}+\sum_{j=0}^{w-1}{\overset{⌢}{\xi }}_{w-1-j}{{\overset{⌢}{\phi }}^{\prime }}_{j}\right)-\mathrm{Pr}L_{b}\sum_{j=0}^{w-1}{\overset{⌢}{f}}_{w-1-j}{\overset{⌢}{\xi }}_{j}^{\prime }.$$

Additionally,

(42) $$\chi_{n}=\left\{ \begin{array}{l} 0,\;\mathrm{if}\;n\leq 1\\ 1,\;\mathrm{if}\;n>1. \end{array}\right.$$

## 2. Results and Discussion

We now deliberate the consequences of the current exploration from the relevant sketched graphical features on velocity, temperature, and concentration profiles.

### 2.1. Velocity Profile

**Figure 3:**
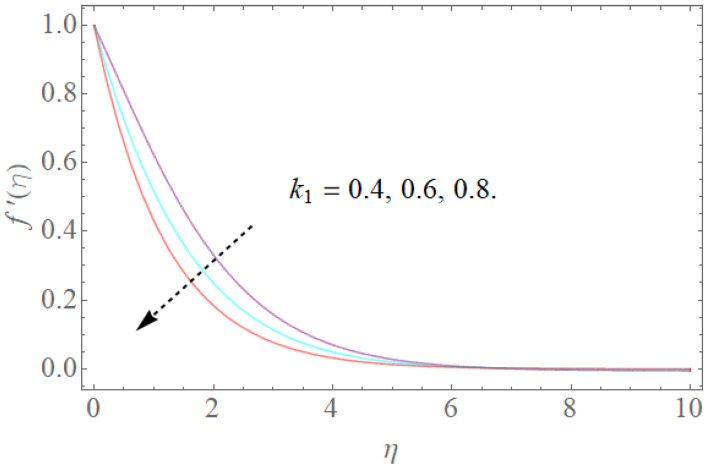
The effect of $k_{1}$ on $f^{\prime }\left(\eta \right)$ when $\beta =1.0,K=1.2,R_{b}=0.4,N_{r}=2.0,\lambda =1.1$.

**Figure 4:**
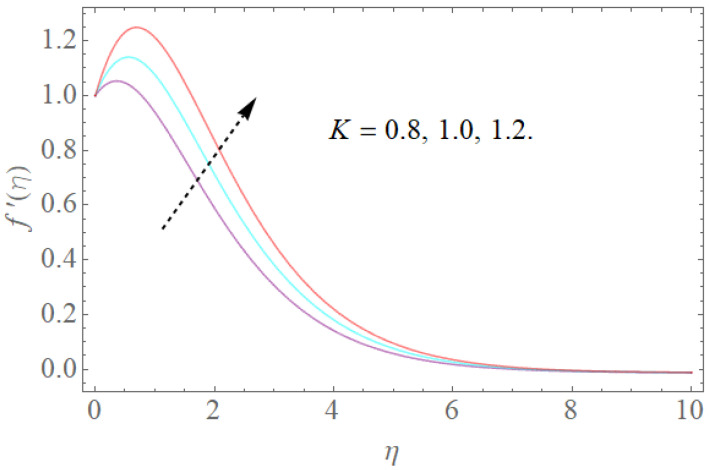
The effect of K on $f^{\prime }\left(\eta \right)$ when $\beta =1.0,k_{1}=1.2,R_{b}=0.4,N_{r}=2.0,\lambda =1.1$.

**Figure 5:**
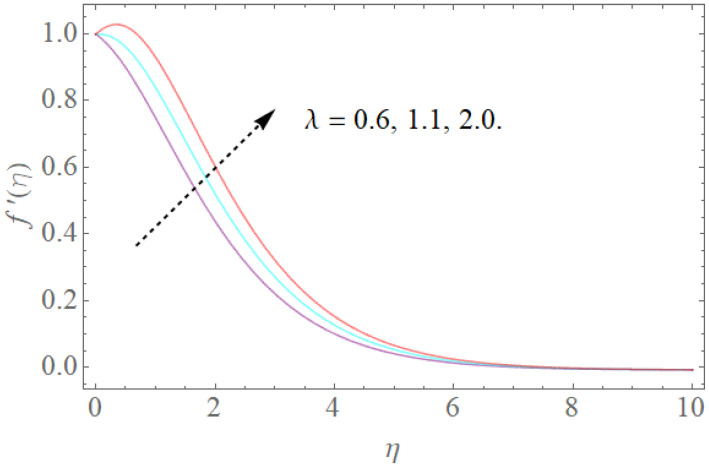
The effect of λ on $f^{\prime }\left(\eta \right)$ when $\beta =1.0,k_{1}=1.2,R_{b}=0.4,N_{r}=2.0$.

The impacts of $\beta ,k_{1},\lambda ,K$ on velocity fields $f^{\prime }\left(\eta \right)$ are demonstrated in Figure 2, Figure 3, Figure 4 and Figure 5. The impacts of the magnetic parameter (β) on the dimensionless velocity field $f^{\prime }\left(\eta \right)$ is seen in Figure 2. It is clear that as (β) increases, $f^{\prime }\left(\eta \right)$ decreases. The velocity profile in the domain decreases as the magnetic parameter (β) is increased, as seen in the sketch. Physically, this happens when the Lorentz force increases as the magnetic parameter increases, resulting in a decrease in liquid velocity, as seen in Figure 2. Figure 3 illustrates how increasing the porosity factor increases the system’s resistance. Physically, this causes a decrease in fluid flow due to increased frictional force. The impacts of the couple-stress parameter K on the velocity field is also seen in Figure 4. The velocity profile increases as the value of the couple-stress parameter K is raised, as can be seen in this graph. For broad values, however, the rise in velocity would be negligible. That is, large values would result in a pure viscous fluid. The effect of λ on $f^{\prime }\left(\eta \right)$ is depicted in Figure 5. It is reasonable to assume that $f^{\prime }\left(\eta \right)$ has higher values of λ based on Figure 5. Enlarging λ induces an enrichment of pliable force, which causes the boundary layer to extend, as seen in Figure 5.

### 2.2. Dimensionless Induced Magnetic Field along x − Direction $g^{\prime }\left(\eta \right)$

**Figure 6:**
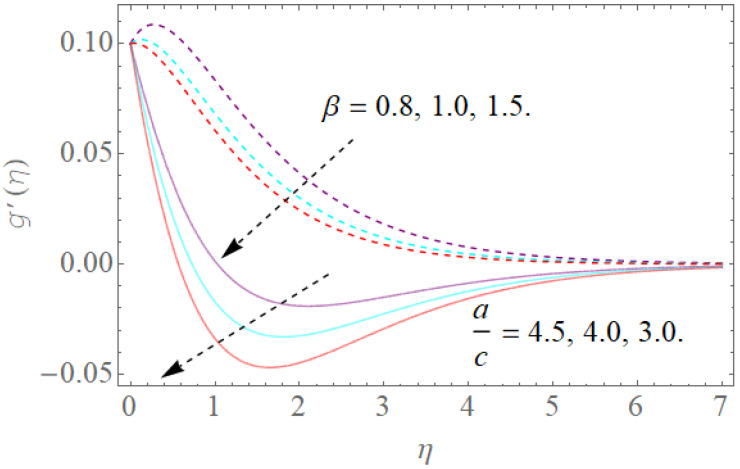
The effect of $\beta ,\frac{a}{c}$ on $g^{\prime }\left(\eta \right)$ when S=1.0,Λ=1.0.

**Figure 7:**
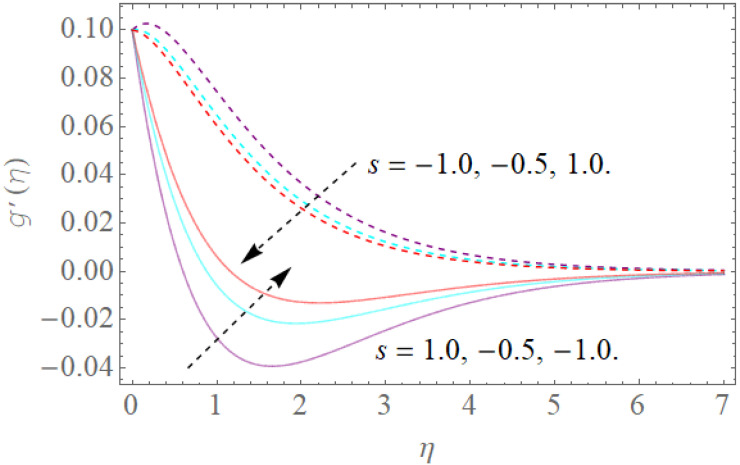
The effect of s>0,s<0 on $g^{\prime }\left(\eta \right)\;$ when $\beta =1.5,\frac{a}{c}=4.0,\Lambda =1.0$.

The impacts of $\beta ,s,\frac{a}{c}$ on the dimensionless-made magnetic field along the x − direction $g^{\prime }\left(\eta \right)$ have been shown in Figure 6 and Figure 7. The impacts of the magnetic parameter (β) and the (a/c) suction or injection parameter on $g^{\prime }\left(\eta \right)$ are shown in Figure 6. It is very clear that by growing the values of (β), $g^{\prime }\left(\eta \right)$ reduces. We notice that an increment in (β) summons an attractive field upgrade (Lorentz power increase) and furthermore dimensionless $g^{\prime }\left(\eta \right)$. Figure 6 also shows the effect of (a/c) on $g^{\prime }\left(\eta \right)$. As a result, a decrease in (a/c) leads to reducing $g^{\prime }\left(\eta \right)$. Figure 7 portrays the impact of the suction/injection parameter over the velocity profile $g^{\prime }\left(\eta \right)$. Increasing the suction or injection parameter initially reduces the $g^{\prime }\left(\eta \right)$ profile, but at that point, as we grow in the direction of the center of the hydrodynamic edge stream, its pattern changes to the reverse direction.

### 2.3. Temperature Profile

**Figure 8:**
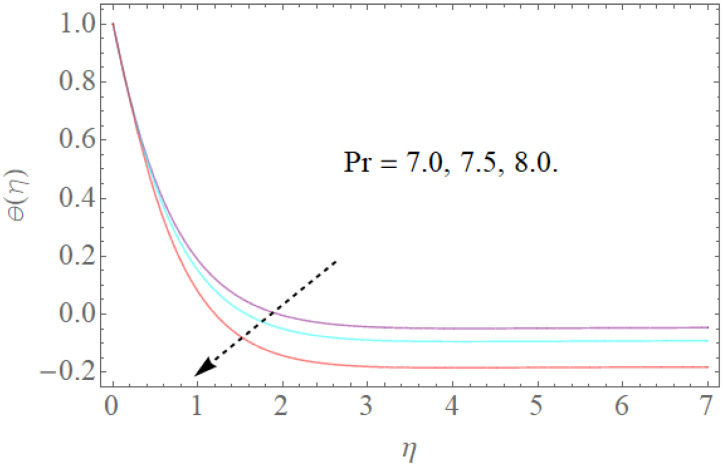
The effect of Pr on θ(η) when $N_{b}=0.3,\alpha =1.0,N_{t}=0.2$.

**Figure 9:**
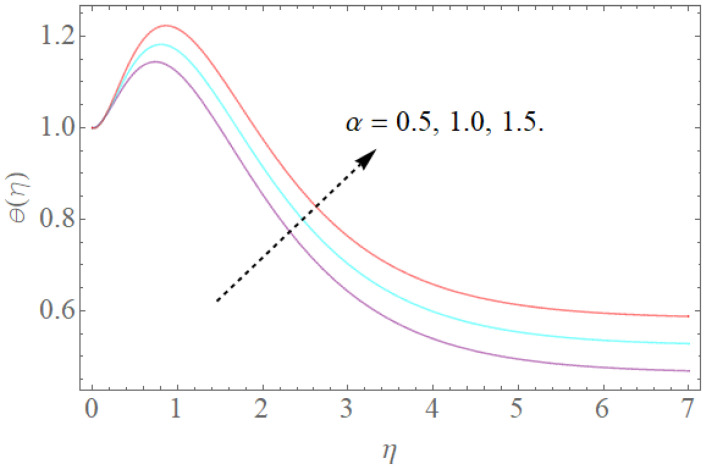
The effect of α on θ(η) when $N_{t}=0.2,\mathrm{Pr}=12,N_{b}=0.5$.

**Figure 10:**
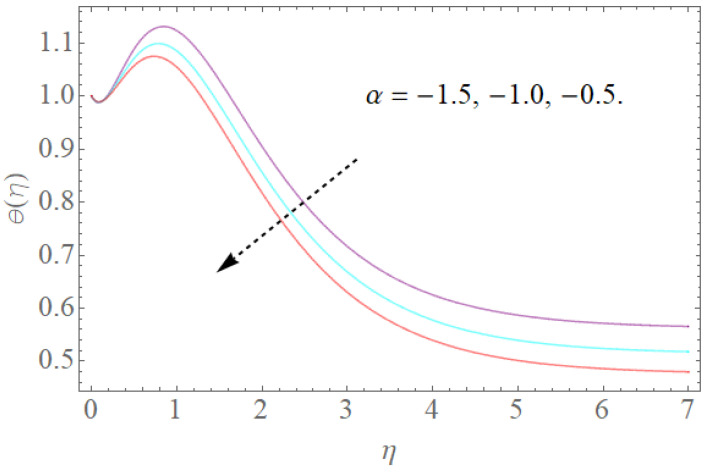
The effect of α on θ(η) when $N_{t}=0.2,\mathrm{Pr}=12,N_{b}=0.5$.

**Figure 11:**
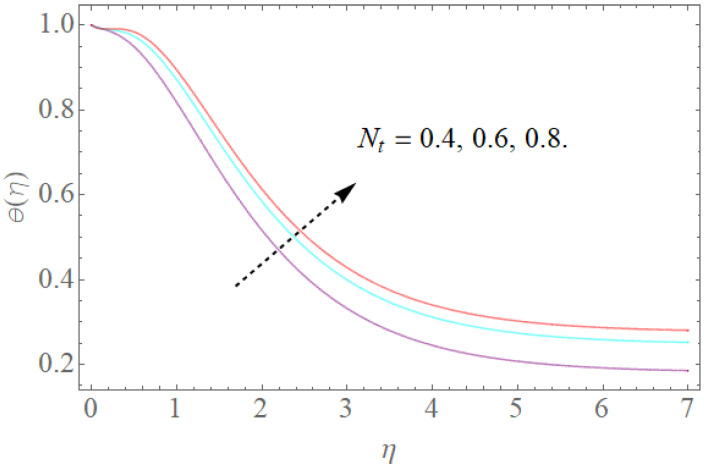
The effect of $N_{t}$ on θ(η) when $N_{b}=0.3,\alpha =1.0,\mathrm{Pr}=12$.

**Figure 12:**
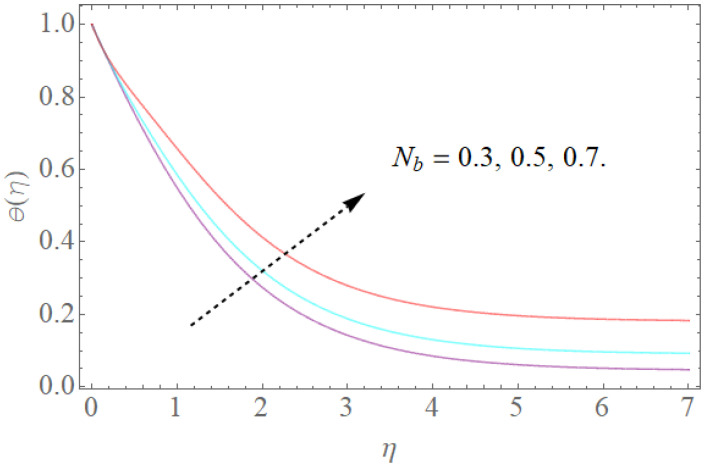
The effect of $N_{b}$ on θ(η) when $N_{t}=0.2,\alpha =1.0,\mathrm{Pr}=7.5,R=0.8$.

Figure 8, Figure 9, Figure 10, Figure 11 and Figure 12 show the effects of physical factors $\mathrm{Pr},N_{b},N_{t},\alpha$ on the temperature distribution θ(η). The results of the Prandtl number on temperature and concentration profiles are seen in Figure 8. The temperature profile indicates a decrease with rising Pr values, as can be seen in Figure 8. Physically, raising the Prandtl number creates a decrease in thermal diffusivity, which is caused by a decrease in the temperature profile. The investigation of the heat source parameter α on θ(η) is depicted in Figure 9. When more heat is applied to the layer, the temperature of fluid particles in the whole domain increases, while in the case of a heat sink, the reverse result occurs, as seen in Figure 10. In the sink situation, the layer loses a lot of heat, dropping the temperature of the fluids. The effect of the thermophoresis boundary $\left(N_{t}\right)$ on the heat profile is shown in Figure 11. The temperature dispersion and the warm limit layer shows expanded conduct for the expansion of the thermophoresis boundary. Truly, the reformist idea of the thermophoretic boundary brings about an expansion of the thermophoretic power inside the liquid system, following improvement in the temperature profile and related limit layer. The effect of the Brownian dispersion boundary $\left(N_{b}\right)$ on the heat profile is shown in Figure 12. For the Brownian movement boundary, truly, the reformist nature warms the actual arrangement. This warming winds up moving nanoparticles from the colder extending sheet district to the quiet liquid locale.

### 2.4. Concentration Profile

**Figure 13:**
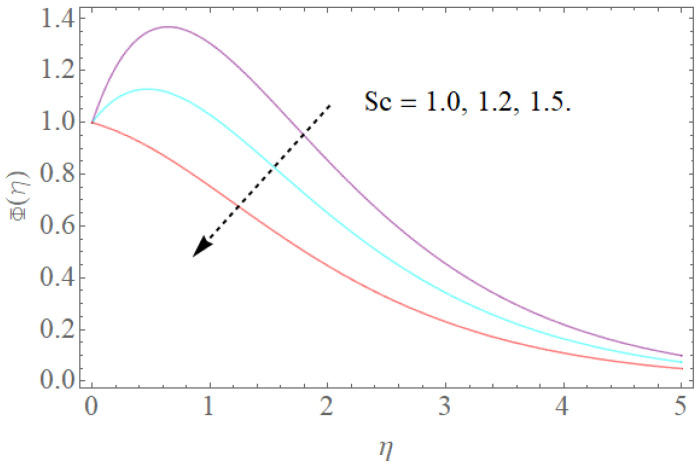
The effect of Sc on Φ(η) when $N_{t}=0.3,N_{b}=0.2$.

**Figure 14:**
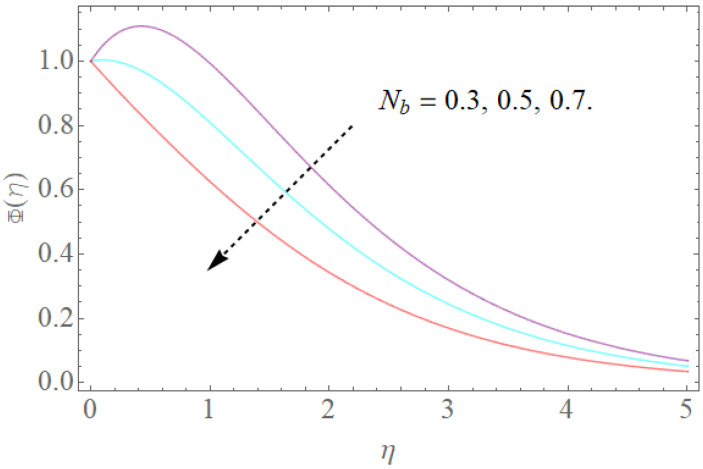
The effect of $N_{b}$ on Φ(η) when $N_{t}=0.3,Sc=1.0$.

**Figure 15:**
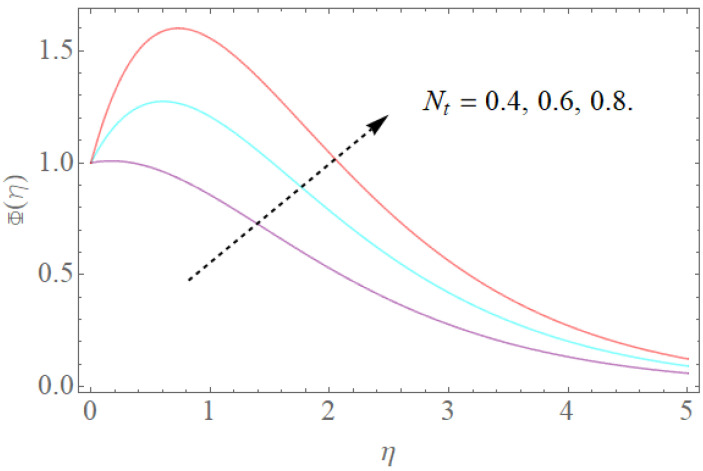
The impact of $N_{t}$ on Φ(η) when $N_{b}=0.3,Sc=1.0$.

The outcome of physical factors $Sc,N_{t},N_{b}$ has been examined for concentration distribution Φ(η) in Figure 13, Figure 14 and Figure 15. The graphical results for Sc are displayed in Figure 13. This is very clear that Sc increases as the concentration profile Φ(η) declines. Therefore, the concentration decreases, Sc and kinematic viscosity physically increase due to the reduction of molecular diffusion. Figure 14 shows that the growth in the parameter of the Brownian motion $N_{b}$ produces the reduction in the concentration profile Φ(η) of the fluid because nanoparticles move from the high concentration region to the region with less concentration. It is obvious that the increase in motion of the nanoparticles produces a high Brownian motion, and this irregular Brownian motion from high concentration areas to lower concentration regions reduces the momentum of the fluid. Figure 15 demonstrates that the escalation in the thermophoresis boundary $N_{t}$ produces the high concentration profile Φ(η) of the stream. These meet with zero at the boundary stream layer. The small variation in the thermophoresis boundary prompts fast movement in the liquid particles, making an abundance of heat energy and prompting a gigantic expansion in the focus dispersion. With an improvement in the calculation of $N_{t}$, Figure 15 indicates a huge expansion in the focus dissemination.

### 2.5. Microorganism Distribution

**Figure 16:**
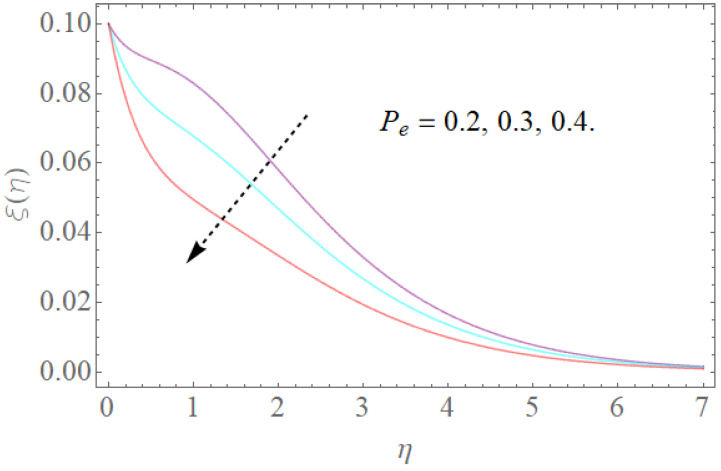
The effect of $P_{e}$ on ξ(η) when $L_{b}=1.5,\sigma_{1}=0.5$.

**Figure 17:**
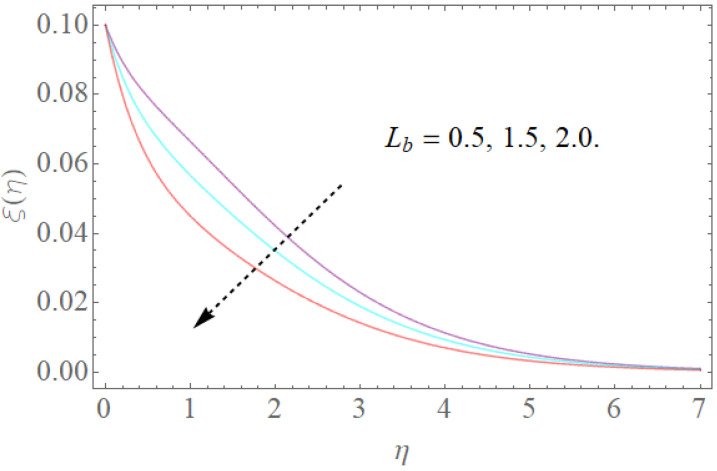
The effect of $L_{b}$ on ξ(η) when $P_{e}=0.4,\sigma_{1}=0.5$.

**Figure 18:**
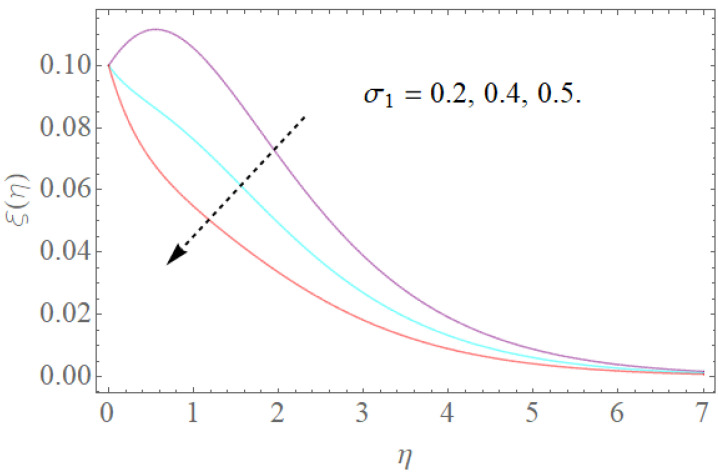
The effect of $\sigma_{1}$ on ξ(η) when $P_{e}=0.4,L_{b}=1.5$.

**Figure 19:**
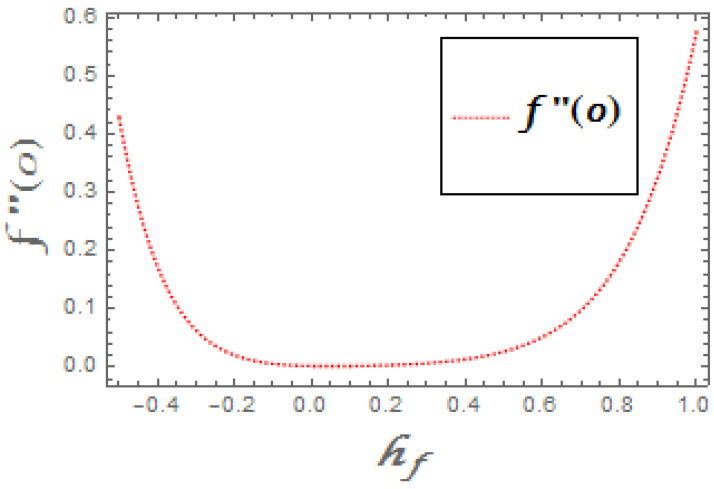
The h-curve graph for $f^{\prime \prime }(0)$.

**Figure 20:**
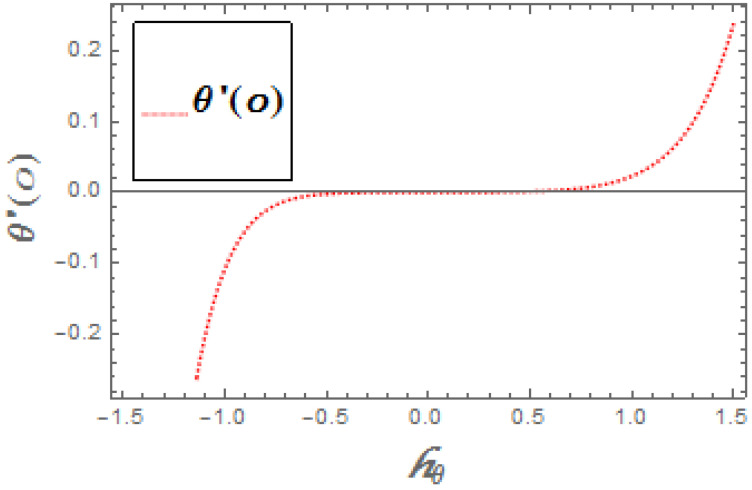
The h-curve graph for $\theta^{\prime }\left(0\right)$.

**Figure 21:**
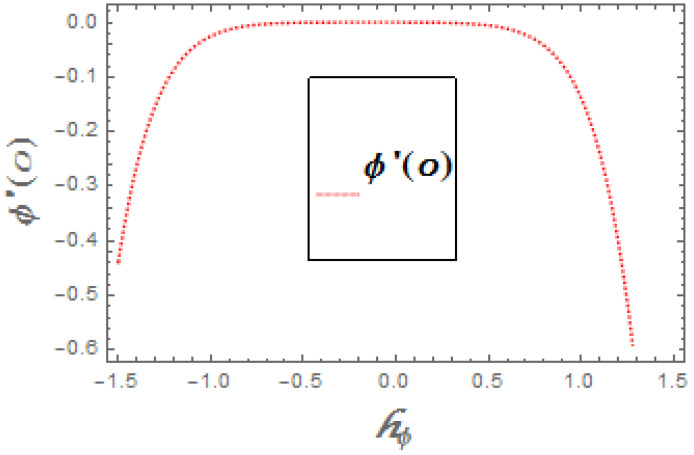
The h-curve graph for $\Phi^{\prime }\left(0\right)$.

**Figure 22:**
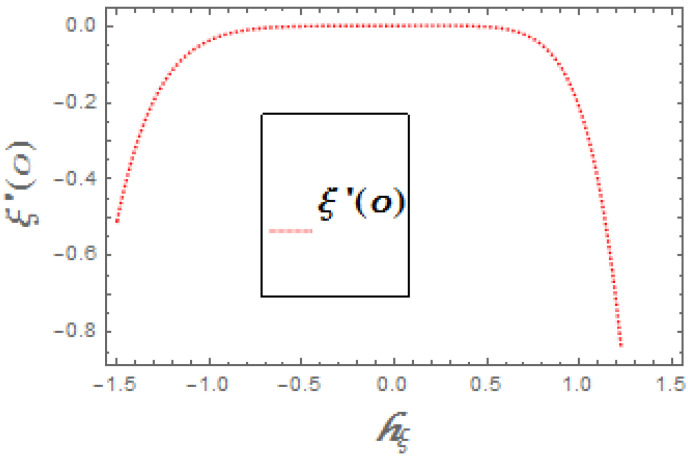
The h-curve graph for $\xi^{\prime }\left(0\right)$.

The portrayed behavior of several estimations of the bioconvection $L_{b}$ and Peclet number in Figure 16 and Figure 17 indicates that improving the bioconvection and Peclet number induces a fast reduction in the thickness for motile microorganisms. That is, the thickness of motile microorganisms was decreased, and to be sure, by reinforcing the bioconvection Lewis number and Peclet number, the decrease in microorganisms’ dispersion was deciphered. This produces the thickness and limit layer thickness slumped for motile microorganisms by raising the worth in $L_{b}$ and $P_{e}$. Figure 18 depicts the effect of the $\sigma_{1}$ on the rising parameters, which slows down the density of motile microorganisms. Figure 19, Figure 20, Figure 21 and Figure 22 show h-curves graphs of $f^{\prime \prime }(0),\theta^{\prime }\left(0\right),\Phi^{\prime }\left(0\right)$, and $\xi^{\prime }\left(0\right)$, respectively.

### 2.6. Tables Discussion

Table 3 shows that $c_{fx}$ increased when the values of $F_{1},k_{1},\beta$ increased. The $c_{fx}$ is decreased when the value of K increased. Table 4 shows that $Nu_{x}$ increased when the values of R,α increased. The $Nu_{x}$ decreased when the values of $\mathrm{Pr},N_{b},N_{t}$ increased. Table 5 shows that Sh increased when the value of Sc increased. The Sh decreased when the values of $N_{b},N_{t}$ increased. Table 6 shows that $Nn_{x}$ increased when the values of $L_{b},\sigma_{1},P_{e}$ increased. Table 7 is the comparison between the ND solves method and HAM method.

## 3. Conclusions

In this exploration, we analyzed the persistent laminar-blended convection of thick viscous and electrically leading sets of nanofluid cross breed Darcy–Forchheimer CuO-Cu/Blood stress stream close to the stagnation-point in the plane past a level permeable extending load up. This is utilized in biomedical fields, for example, the miniature roundabout framework’s stream elements and particularly in the inventory of medications. The fundamental partial differential equations (PDEs) are modified to a bunch of dimensionless ordinary differential equations (ODEs) with the assistance of reasonable comparability variables. These coupled ODEs are then solved by utilizing the Homotopy Analysis Method (HAM).

After detail study of the work, that the following conclusions were observed:

- When increasing the value of the magnetic field parameter, the porosity factor velocity profiles decrease.
- The velocity profile rises with a rise in the value of couple-stress parameter K.
- The velocity profile displays a rising feature for greater values of λ.
- With the enhancement of the strength of the thermophoresis parameter and the Brownian diffusion parameter, the temperature profile increases.
- With the enhancement of the strength of the heat source (s>0), the fluid temperature increases; on the other hand, an increase in the heat sink strength (s<0) decreases the temperature.
- By increasing the value of the Prandtl factor, the fluid temperature decreases.
- With an increase in the strength of the thermophoresis parameter and Brownian diffusion parameter, both have reverse impact on the concentration profile.
- With an increase in the value of Sc, the concentration profile decreases.
- The Φ(η) portrayed a decreasing tendency with the rising number of $L_{e}$.
- The density of the moving microorganisms inside the fluid reduces for large values of $P_{e}$.
- The density number reduces to increase $L_{b}$.

## Figures and Tables

**Table 1:** Nanoparticles and primary base fluid have the following thermophysical properties [8].

Thermophysical Properties	Fluid Phase (Blood)	Copper Oxide (CuO)	Copper (Cu)
*c_p_* (J/kgK)	3594	533	385
*ρ* (kg/m^3^)	1063	6500	8933
*k* (w/mK)	0.492	17.65	400
*β* × 105 (L/K)	0.18	1.8	1.67
Particles size (nm)	-	29	5–25

**Table 3:** The effect on the Skin friction Res12Cf=(1−w1ρ1+w2ρ2w1ρ1+w2ρ2+wfρf)f″(0), with the microorganism of different physical parameters. The impact of various physical parameters over.

*F*_1_	*k*_1_	*β*	*K*	*N_r_*	*λ*	$\left(1-\frac{\frac{w_{1}}{\rho_{1}}+\frac{w_{2}}{\rho_{2}}}{\frac{w_{1}}{\rho_{1}}+\frac{w_{2}}{\rho_{2}}+\frac{w_{f}}{\rho_{f}}}\right)f^{\prime \prime }\left(0\right),$
0.3	0.3	0.5	0.4	1.0	0.6	2.5313646
0.5						2.6768415
0.7						2.8147213
	0.3					1.2310745
	0.4					1.3324613
	0.6					1.4345015
		0.5				0.6156063
		1.0				0.8367109
		1.5				1.1348550
			0.4			3.5157305
			0.8			3.1516109
			1.0			3.0357126
				1.0		2.9461262
				2.0		3.5240782
				3.0		3.7180281
					0.6	2.0146849
					1.0	1.6130745
					1.5	1.2154047

**Table 4:** The effect on the Nusselt number $-\frac{k_{hnf}}{k_{f}}\left(1+\frac{4}{3}R\right)\theta^{\prime }\left(0\right)$ with the microorganism of different physical parameters.

α	Pr	*N_t_*	*N_b_*	$-\frac{k_{hnf}}{k_{f}}\left(1+\frac{4}{3}R\right)\theta^{\prime }\left(0\right)$
−0.5	8.5	0.7	0.5	3.6374583
−0.5				3.4584563
−1.0				3.1524204
	8.5			2.3482139
	9.5			2.6434058
	10.5			2.8464381
		0.2		2.7125861
		0.4		2.5439308
		0.6		2.1643615
			0.5	0.9614792
			0.7	1.4739615
			0.9	1.7905246

**Table 5:** The effect on the Sherwood number Rex12Shx=−ϕ′(0). with the microorganism of different physical parameters.

*N_t_*	*N_b_*	*Sc*	$-\phi^{\prime }\left(0\right)$
0.2	0.3	1.0	1.9352426
0.4			2.2568761
0.6			2.5917425
	0.3		1.2518701
	0.7		1.4507817
	0.9		1.7591572
		1.0	1.4523497
		1.3	0.5248979
		1.5	0.1935468

**Table 6:** The effect on the motile NnxRex−1/2=−ξ′(0) with the microorganism of different physical parameters.

*P*_*e*_	*σ*_1_	*L*_*b*_	$-\xi^{\prime }(0)$
0.2	0.3	0.5	3.3467521
0.3			3.2674302
0.4			3.1459609
	0.3		4.2670293
	0.4		4.1523549
	0.5		4.0795164
		0.5	2.3895186
		1.5	2.1384597
		2.0	2.0634175

**Table 7:** A comparison between ND solves and HAM methods.

*η*	*f*′(*η*)		*θ*(*η*)		*ϕ*(*η*)		*ξ*(*η*)	
	NDSolve	HAM	NDSolve	HAM	NDSolve	HAM	NDSolve	HAM
0	$\begin{array}{c} 1.000 \end{array}$	$\begin{array}{c} 1.000 \end{array}$	1.000	1.000	1.000	1.000	1.000	1.000
0.5	1.267861	1.267872	$\begin{array}{c} 1.034338 \end{array}$	$\begin{array}{c} 1.034340 \end{array}$	0.583358	0.583360	0.8434886	0.8434888
1.0	0.838512	0.838518	1.021331	1.021333	0.307213	0.307215	0.7330826	0.7330829
1.5	−0.03184	−0.03188	0.965956	0.965958	0.143647	0.143650	0.6444098	0.6444100
2.0	−0.82835	−0.82837	0.874652	0.874654	0.057064	0.057066	0.5616697	0.5616700
2.5	−1.08151	−1.08153	0.754801	0.754804	0.016656	0.016658	0.4770605	0.4770600
3.0	−0.64058	−0.64060	$\begin{array}{c} 0.614284 \end{array}$	$\begin{array}{c} 0.614286 \end{array}$	0.001988	0.001990	0.3896790	0.3896793
3.5	0.2365287	0.2365289	0.461086	0.461088	$\begin{array}{c} 0.000735 \end{array}$	$\begin{array}{c} 0.000736 \end{array}$	0.3012565	0.3012567
4.0	1.0290494	1.0290496	0.302960	0.302963	$\begin{array}{c} 0.004461 \end{array}$	$\begin{array}{c} 0.004460 \end{array}$	0.2111275	0.2111277
4.5	$\begin{array}{c} 1.2528515 \end{array}$	$\begin{array}{c} 1.2528517 \end{array}$	0.147122	0.147125	0.006107	0.006110	0.1139330	0.1139333
5.0	$\begin{array}{c} 0.7499995 \end{array}$	$\begin{array}{c} 0.7499997 \end{array}$	0	0	0	0	0	0

## Data Availability

There is no data availability regarding this research.

## Abbreviations

Symbol	Discription
*u_w_*	stretching velocity *(*m/s*)*
u and v	velocity components along *x-* and *y*-axes (m/s)
*H*_1_ and *H*_2_	magnetic *fi*eld components along *x-* and *y*-axes
*η_0_*	Magnetic diffusivity
*β*	Magnetic field parameter
*β*	volumetric thermal expansion coef*fi*cient
*μ_hnf_*	viscosity of hybrid nano*fl*uid
*k_hnf_*	thermal conductivity of hybrid nano*fl*uid
*Sh*	Sherwood number
*ϕ*	nanoparticle volume fraction
*k**	mean absorption coefficient
*s*	suction or injection parameter
*w_1_, w_2_*	the first nanoparticle, the second nanoparticle
*λ*	mixed convection or buoyancy parameter
*μ_e_*	Magnetic permeability
*α*	heat source parameter
*Sc*	Schmidt number
*C_f_*	Skin friction coefficient
*ρ_S_*	density of nanoparticles
*C_w_*	Surface concentration
*T*	Temperature of the fluid (K)
*C*	Concentration of the fluid
*Nn*	Concentration of the fluid
*V_0_*	suction/injection velocity
*F_1_*	Inertia coefficient
*θ*	Dimentionless temperature (-)
*ρ_f_*	Density (kg × m^−3^)
*f’*	Dimentionless velocity (-)
*R_b_*	Bịoconvectịon Rayleigh number
*μ_f_*	Dynamic viscosity (kg × m^−1^ × s^−1^)
*P_e_*	Bioconvection Peclet number
*T∞*	Ambient temperature (K)
*T_0_*	Reference temperature (K)
*k*	Dimensional permeability
*g*	acceleration due to gravity *(*m/s^2^)
*λ_1_*	diffusive constant parameter
*Q*	volumetric rate of heat generation/absorption
*ρ_hnf_*	Density of hybrid nanofluid (kg × m^−3^)
*(ρc_p_)_hnf_*	volumetric heat capacity of hybrid nano*fl*uid (m^2^ × s^−2^ × K^−1^)
*Re_x_*	Reynolds number
*ϕ_1_, ϕ_2_*	volume fraction of first and second nanoparticles
*(c_p_)_s_*	specific heat at constant pressure of nanoparticles (m^2^ × s^−2^ × K^−1^)
*w_f_*	base fluid masses
*k_1_*	Non-dimensional permeability parameter
*N_t_*	thermophoretic parameter
*Λ*	Reciprocal magnetic prandtl number
*K*	coupled stress parameter
*Nu_x_*	Nusselt number
*Pr*	Prandtl number
*T_w_*	Surface temperature (K)
*N_b_*	Brownian motion parameter
*l*	characteristic length (m)
*σ*	concentration difference parameter
*Gr*	Grashof number
*k*	Thermal conductivity (W × m^−1^ × K^−1^)
*Nr*	Buoyancy ratio parameter
*Φ*	Dimentionless concentration (-)
*η*	Similarity variable
*ν_f_*	Kinematic viscosity (m^2^ × s^−1^)
*(ρc)_p_*	Effective heat capacity of nanoparticles (m^2^ × s^−2^ × K^−1^)
*L_b_*	Bioconvection Lewis number
